# Laparoscopic versus open emergent colectomy for ischemic colitis: a propensity score-matched comparison

**DOI:** 10.1186/s13017-022-00458-4

**Published:** 2022-10-13

**Authors:** Yi-Chang Chen, Yuan-Yao Tsai, Sheng-Chi Chang, Hung-Chang Chen, Tao-Wei Ke, Abe Fingerhut, William Tzu-Liang Chen

**Affiliations:** 1 Institute of Medicine, Chung Shan Medical University, Taichung, Taiwan, ROC; 2 Department of Colorectal Surgery, China Medical University Hospital, Taichung, Taiwan, ROC; 3 Department of General Surgery, Ruijin Hospital, Shanghai Jiao Tong University School of Medicine, Shanghai, China; 4 Section for Surgical Research, Department of Surgery, Medical University of Graz, Graz, Austria; 5 Department of Colorectal Surgery, China Medical University Hsinchu Hospital, No. 199, Sec.1, Xinglong RD, Zhubei City, 30272 Hsinchu County Taiwan, ROC

**Keywords:** Laparoscopy, Emergent colectomy, Ischemic colitis

## Abstract

**Introduction:**

Laparoscopic colectomy is rarely performed for ischemic colitis. The aim of this propensity score-matched study was to compare preoperative characteristics, intraoperative details and short-term outcomes for emergent laparoscopic colectomy versus the traditional open approach for patients with ischemic colitis.

**Methods:**

Retrospective review of 96 patients who underwent emergent colectomy for ischemic colitis between January 2011 and December 2020 (39 via laparoscopy, 57 via laparotomy) was performed. We compared short-term outcomes after using a one-to-one ratio and nearest-neighbor propensity score matching to obtain similar preoperative and intraoperative parameters in each group.

**Results:**

Patients in the open group experienced more surgical site complications (52.6% vs. 23.0%, *p* = 0.004), more intra-abdominal abscesses (47.3% vs. 17.9%, *p* = 0.003), longer need for ventilator support (20 days vs. 0 days, *p* < 0.001), more major complications (77.2% vs. 43.5%, *p* = 0.001), higher mortality (49.1% vs. 20.5%, *p* = 0.004), and longer hospital stay (32 days vs. 19 days, *p* = 0.001). After propensity score matching (31 patients in each group), patients undergoing open (vs. laparoscopy) had more surgical site complications (45.1% vs. 19.4%, *p* = 0.030) and required longer ventilator support (14 vs. 3 days, *p* = 0.039). After multivariate analysis, Charlson Comorbidity Index (*p* = 0.024), APACHE II score (*p* = 0.001), and Favier’s classification (*p* = 0.023) were independent predictors of mortality.

**Conclusions:**

Laparoscopic emergent colectomy for ischemic colitis is feasible and is associated with fewer surgical site complications and better respiratory function, compared to the open approach.

## Introduction

Ischemic colitis (IC), characterized by insufficient blood supply and oxygen delivery to the colon, is the most common form of bowel ischemia [1]; the incidence of IC ranges from 4.5 to 44 cases per 100,000 person years [2]. The severity of IC depends on the degree of parietal involvement, ranging from superficial mucosa inflammation, amenable to conservative or medical treatment, to full-thickness transmural necrosis, a life-threatening condition that requires surgery [3–6]. The mortality rate after surgery is high, up to 60% [7], usually related to transmural bowel necrosis and patient comorbidity [8].

The benefits of laparoscopy have been well described for elective [9, 10] and more recently, for emergent colorectal surgery [11–14]. However, publications on the laparoscopic approach for IC are rare; most patients undergo laparotomy [15–18]. The aim of this study is to report the short-term outcomes of laparoscopic management of patients with IC, compared to open colectomy, in propensity score-matched groups.

## Patients and methods

**Fig. 1:**
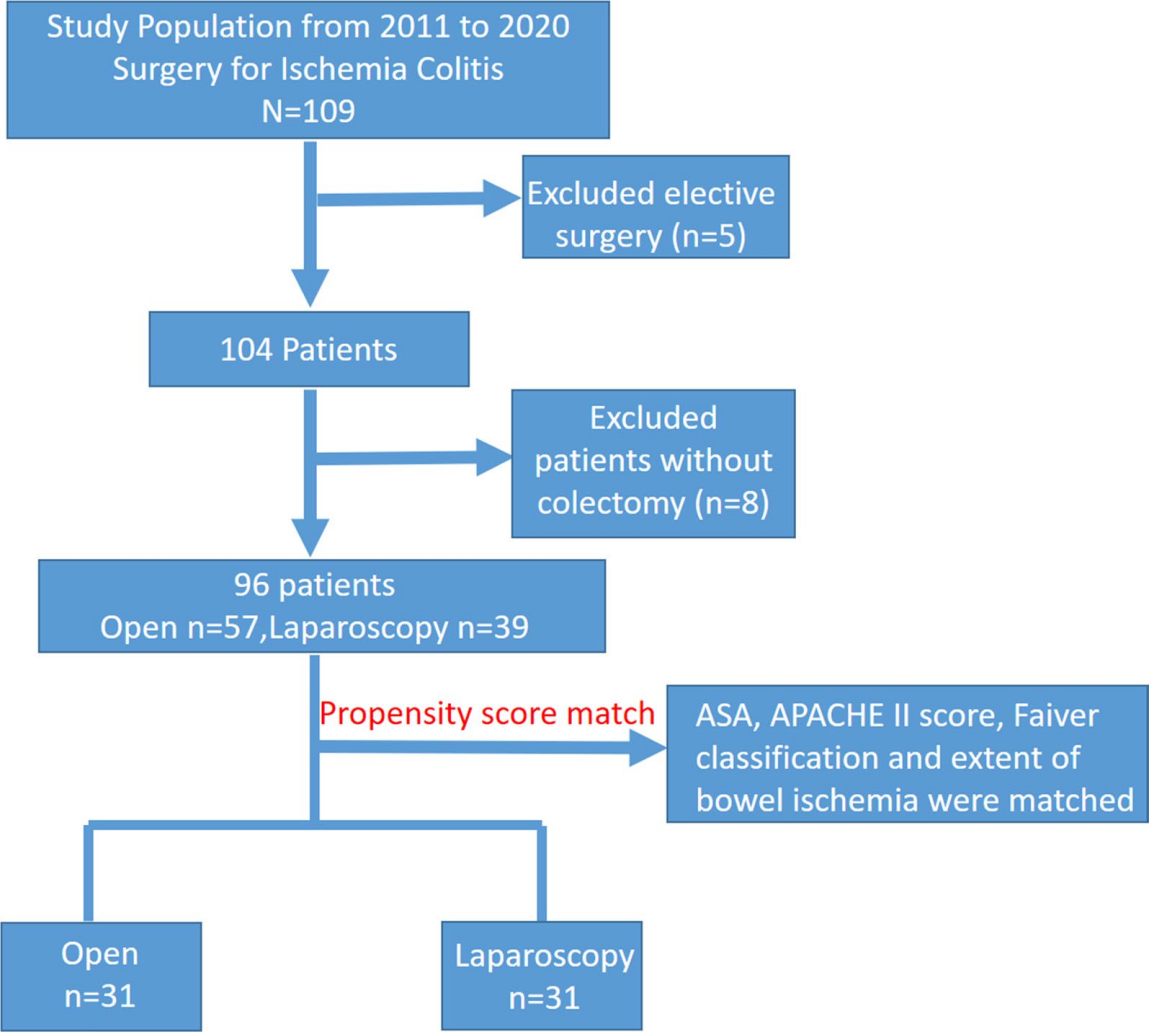
Flowchart of study population

From January 2011 to December 2020, all consecutive patients in China Medical Hospital (Taichung, Taiwan) who underwent emergent surgery for IC confirmed by pathology reports were included in this study (Fig. 1). The study was approved by the departmental and institutional ethical committees of the China Medical University Hospital, Taichung, Taiwan (Registered NO: CMUH110-REC3-067). Baseline characteristics, laboratory, operative, postoperative outcome data were retrospectively reviewed and analyzed. Indications for the minimal access approach depended on surgeon discretion. Patients who initially underwent laparoscopy but who were converted to open surgery were analyzed in the laparoscopic group (intention-to-treat). The extent of colon resection, the performance of anastomosis, and/or stoma was determined on a case-by-case basis, according to surgeons’ judgment, degree of bowel ischemia, and patient clinical status. We categorized ischemia according to Favier’s classification: type I (transient and mild mucosal ischemia), type II (mucosal and muscularis ischemia, generally considered reversible but possibly linked to multiple organ failure), or type III (nonreversible transmural ischemia) [3].

The Charlson Comorbidity Index (CCI) was calculated for each patient [19]. The Acute Physiology and Chronic Health Evaluation II (APACHE II) score was used to evaluate severity of sepsis before operation [20]. Delayed surgery was defined as surgical intervention performed ≥ 3 days after the initial symptoms appeared [21]. The etiology of IC was individualized as postoperative IC (within 15 days postoperative) or not, and among the latter, as occlusive and non-occlusive [17, 22]. Duration of ventilator support and hospital stay were counted starting from the day of emergency colectomy for IC. Deceased patients were excluded from statistical analyses of these two parameters. Morbidity and mortality were recorded at 30 days after the second operation or during the same hospital stay if the patient was hospitalized longer. Complications were graded using the Clavien–Dindo classification [23]. Major complications were defined as grade III or greater. Intra-abdominal or deep surgical site abscess was defined as abscess formations visualized on computerized tomography (CT) scan. Prolonged ileus was defined as non-emission of flatus ≥ 7 days [24].

### Surgical technique of laparoscopic colectomy for ischemic colitis

**Fig. 2:**
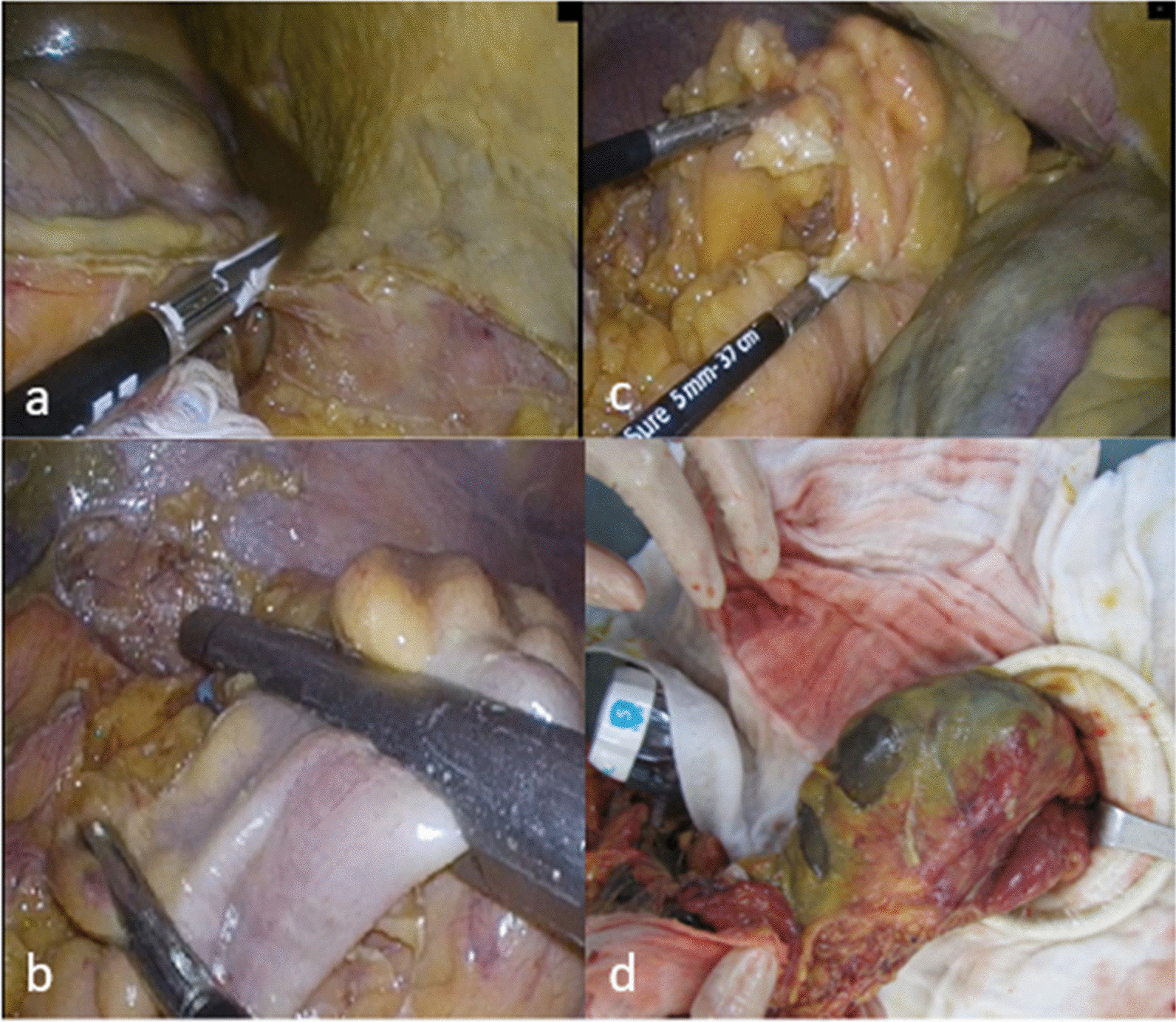
Surgical steps of emergent laparoscopic colectomy for ischemia colitis. **a** Colonic mobilization from lateral side; **b** Endo-GIA was applied at distal and proximal edges of the ischemic colon; **c** Mesentery division with Ligasure; and **d** Specimen was extracted via the umbilicus surgical site or planned stoma site

All patients underwent skin prep and were draped for surgery in the lithotomy position. The first 12 mm trocar was inserted peri-umbilically by the open method. After examination of the entire abdomen, the extent of IC was confirmed and additional trocars were inserted: two on the right and two on the left. The colon was mobilized lateral to medial and then divided proximally and distally with endo-GIA staplers to remove the ischemic segment (Fig. 2). The corresponding mesentery transection was performed using Ligasure (Fig. 2). The ischemic colonic specimen was extracted via the umbilical surgical site or planned stoma site (Fig. 2). Primary anastomosis or Hartmann’s procedure was performed depending on patient clinical status and surgeon experience. Finally, the entire abdomen was abundantly lavaged and an abdominal drain placed.

Categorical data are presented as percentages, and continuous data are expressed as median (with range). Fisher’s exact test or chi-squared test was used for categorical data. Student’s *t* test was used for normally distributed metric data and Mann–Whitney *U* test for non-normally distributed data. A propensity score analysis was performed to obtain a one-to-one match by using the nearest-neighbor matching method. Multiple logistic regression was used to derive propensity scores regarding the probability that a patient would undergo a laparoscopic or open method. Patients in whom the propensity score was not applicable were excluded from further analysis. The following covariates were matched: ASA, APACHE II score, Favier classification, and extent of bowel ischemia. Variables with *p* values < 0.05 from univariate analysis were included in a stepwise multivariate logistic regression analysis to evaluate the probability of mortality. Receiver operating characteristic (ROC) curve analysis was performed to identify the optimal cutoff value of APACHE II score for mortality according to the area under the ROC curve (AUC). All statistical analyses were performed with SPSS for Windows (version 25.0; IBM-SPSS Inc., Armonk, NY).

## Results

From January 2011 to December 2020, 96 patients underwent emergent colectomy for IC, 39 via laparoscopy, 57 via laparotomy (Fig. 1). Before propensity score matching, statistically significant differences between laparoscopic and open groups included higher ASA (ASA 4: 64.9% vs. 38.4%, *p* = 0.024) and higher APACHE II score (19 vs. 13, *p* < 0.001) for the open group compared to the laparoscopic group, respectively (Table 1). There was no statistically significant difference in CCI (open vs. laparoscopic: 5 vs. 6, *p* = 0.772). All patients were Favier II or III: There were more patients in the open group who were Favier III (75.4% vs. 51.3%, *p* = 0.014) and had total colonic ischemia (19.4% vs. 5.1%, *p* = 0.004) (vs. laparoscopic group) (Table 2). There was no statistically significant difference in the proportion of patients with bowel perforation (open vs. laparoscopic: 45.6% vs. 41%, *p* = 0.656). After propensity score matching, none of the variables were statistically significantly different in the two (open and laparoscopic) groups of 31 patients each.

**Table 1:** Patient characteristics

	Unmatched			Matched		
	Open (*n* = 57)	Laparoscopy (*n* = 39)	*P* value	Open (*n* = 31)	Laparoscopy (*n* = 31)	*P* value
Year of surgery			0.227			0.127
2011–2015	32 (56.1%)	17 (43.5%)		18 (58.0%)	12 (38.7%)	
2016–2020	25 (43.9%)	22 (56.5%)		13 (42.0%)	19 (61.3%)	
Age (years)	67 (21–89)	70 (22–94)	0.588	66 (21–89)	71 (22–94)	0.446
Gender			0.811			0.798
Male	22 (38.5%)	16 (41%)		14 (45.1%)	13 (41.9%)	
Female	35 (61.5%)	23 (59%)		17 (54.8%)	18 (58.0%)	
BMI (kg/m^2^)	24.3 (14.8–38.2)	22.9 (17.5–30.8)	0.622	24.3 (14.8–30.9)	22.9 (17.5–30.5)	0.354
ASA			0.024			0.778
2	1 (1.8%)	4 (10.2%)		1 (3.2%)	2 (6.4%)	
3	17 (29.8%)	20 (51.2%)		14 (45.1%)	15 (48.3%)	
4	37 (64.9%)	15 (38.4%)		16 (51.7%)	14 (45.3%)	
5	2 (3.5%)	0		0	0	
CCI	5 (0–11)	6 (0–11)	0.772	5 (0–10)	6 (0–11)	0.208
HTN	36 (63.1%)	24 (61.5%)	0.478	18 (58%)	20 (64.5%)	
DM	20 (35%)	14 (35.8%)	0.935	9 (29%)	11 (35.4%)	
CKD	15 (26.3%)	7 (17.9%)	0.338	2 (6.4%)	3 (9.6%)	
CAD	11 (19.3%)	10 (25.6%)	0.460	5 (16.1%)	9 (29.0%)	
CVA	4 (7%)	6 (15.3%)	0.187	3 (9.6%)	6 (19.3%)	
Malignancy	21 (36.8%)	15 (28.4%)	0.872	14 (45.1%)	11 (25.4%)	
APACHE II	19 (6–32)	13 (6–30)	< 0.001	15 (6–28)	15 (6–30)	0.522
Surgical delay (≥ 3 days)	34 (59.6%)	17 (43.5%)	0.121	21 (67.7%)	15 (48.3%)	0.123
Etiology of IC			0.872			0.421
Postoperative IC	21 (36.8%)	15 (38.4%)		9 (29.0%)	12 (38.7%)	
Vascular surgery	13 (22.8%)	4 (10.3%)		5 (16.1%)	4 (12.9%)	
Other surgery	8 (14.0%)	11 (28.2%)		4 (12.9%)	8 (25.8%)	
Other etiologies	36 (63.1%)	24 (61.5%)		22 (70.9%)	19 (61.3%)	
Occlusive	16 (28.0%)	11 (28.2%)		9 (29.0%)	8 (25.8%)	
Non occlusive	20 (25.0%)	13 (33.3%)		12 (38.7%)	11 (35.5%)	

BMI, Body mass index; ASA, American Society of Anesthesiologists; CCI, Charlson Comorbidity Index; HTN, hypertension; DM, diabetes mellitus; CKD, chronic kidney disease; CAD, coronary artery disease; CVA, cerebrovascular accident; and APACHE, Acute Physiology and Chronic Health Evaluation

**Table 2:** Perioperative details

	Unmatched			Matched		
	Open (*n* = 57)	Laparoscopy (*n* = 39)	*P* value	Open (*n* = 31)	Laparoscopy (*n* = 31)	*P* value
Favier’s classification			0.014			0.602
1	0	0		0	0	
2	14 (24.6%)	19 (48.7%)		11 (35.4%)	13 (41.9%)	
3	43 (75.4%)	20 (51.3%)		20 (64.6%)	18 (58.1%)	
Perforation	26 (45.6%)	16 (41.0%)	0.656	13 (41.9%)	10 (32.2%)	0.430
Ischemia site			0.004			0.175
Segmental	15 (26.3%)	20 (51.3%)		15 (48.4%)	17 (54.8%)	
Right colon	14 (24.5%)	2 (5.1%)		7 (22.6%)	2 (6.5%)	
Left colon	17 (29.8%)	15 (38.5%)		9 (29.0%)	10 (32.3%)	
Total	11 (19.4%)	2 (5.1%)		0	2 (6.4%)	
Small bowel ischemia	9 (15.8%)	8 (20.5%)	0.512	6 (19.4%)	8 (25.8%)	0.497
Surgical procedure			0.014			0.250
Hartmann	52 (91.2%)	29 (74.4%)		27 (87.0%)	24 (77.4%)	
Anastomosis with stoma	1 (1.8%)	0		1 (3.2%)	0	
Double barrel stoma	3 (6.0%)	10 (25.6%)		3 (9.8%)	7 (22.6%)	
Blood loss (cc)	100 (30–3500)	50 (30–1600)	0.326	100 (30–1000)	50 (30–1699)	0.469
Conversion	–	9 (23.0%)		–	7 (22.5%)	
Operation time (min)	200 (120–360)	180 (120–600)	0.660	180 (120–360)	180 (120–420)	0.693

Perioperative results and short-term morbidity are summarized in Table 3. Before propensity score matching, patients in the open group had more surgical site complications (52.6% vs. 23.0%, *p* = 0.004), higher proportion of intra-abdominal abscess (47.3% vs. 17.9%, *p* = 0.003), longer ventilator support (20 days vs. 0 days, *p* < 0.001), more major complications (77.2% vs. 43.5%, *p* = 0.001), higher mortality (49.1% vs. 20.5%, *p* = 0.004), and longer hospital stay (32 days vs. 19 days, *p* = 0.001). After propensity score matching, patients undergoing open (vs. laparoscopy) had more surgical site complications (45.1% vs. 19.4%, *p* = 0.030) and required longer ventilator support (14 days vs. 3 days, *p* = 0.039), whereas there was no statistically significant difference in rates of major complications (64.5% vs. 48.3%, *p* = 0.200) or mortality (38.7% vs. 25.8%, *p* = 0.277). However, there was a trend toward fewer intra-abdominal abscess (38.7% vs. 19.4%, *p* = 0.093) and shorter hospital stay (32 days vs. 20 days, *p* = 0.061).

**Table 3:** Postoperative outcome

	Unmatched			Matched		
	Open (*n* = 57)	Laparoscopy (*n* = 39)	*P* value	Open (*n* = 31)	Laparoscopy (*n* = 31)	*P* value
Surgical site complication	30 (52.6%)	9 (23.0%)	0.004	14 (45.1%)	6 (19.4%)	0.030
Prolonged ileus (%)	21 (36.8%)	8 (20.5%)	0.087	11 (35.4%)	9 (29.0%)	0.587
Intra-abdominal abscess	27 (47.3%)	7 (17.9%)	0.003	12 (38.7%)	6 (19.4%)	0.093
CVA	5 (8.8%)	3 (7.7%)	0.851	2 (6.4%)	2 (6.4%)	0.100
Heart	13 (22.8%)	4 (10.3%)	0.114	4 (12.9%)	3 (9.6%)	0.688
Kidney	17 (29.8%)	4 (10.3%)	0.023	4 (12.9%)	2 (6.4%)	0.390
Ventilator support (days)	20 (0–118)	0 (0–40)	< 0.001	14 (0–118)	3 (0–40)	0.039
Reoperation	14 (24.6%)	8 (20.5%)	0.643	5 (16.1%)	6 (19.4%)	0.740
Ischemia	4 (7.0%)	3 (7.7%)		1 (3.2%)	3 (9.6%)	
Bowel perforation	3 (5.3%)	2 (5.1%)		1 (3.2%)	1 (3.2%)	
Bleeding	1 (1.8%)	1 (2.6%)		0	1 (3.2%)	
Surgical site dehiscence	6 (10.5%)	2 (5.1%)		3 (9.6%)	1 (3.2%)	
PAD	7 (12.3%)	1 (2.6%)	0.091	4 (12.9%)	0	0.039
Major complications	44 (77.2%)	17 (43.5%)	0.001	20 (64.5%)	15 (48.3%)	0.200
Mortality	28 (49.1%)	8 (20.5%)	0.004	12 (38.7%)	8 (25.8%)	0.277
Hospital stay (days)	32 (4–139)	19 (11–75)	0.001	32 (19–139)	20 (11–75)	0.061

CVA, Cerebrovascular accident; PAD, percutaneous abscess drainage

**Fig. 3:**
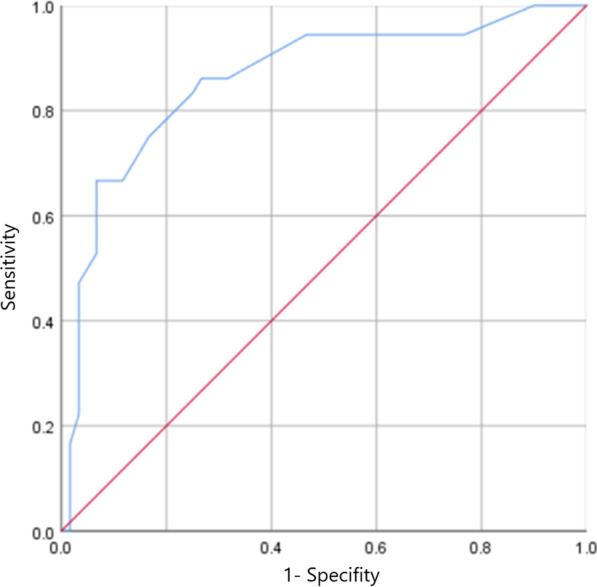
ROC curve for APACHE II score

In univariate analysis, ASA class (*p* = 0.005), CCI (*p* = 0.009), APACHE II score (*p* < 0.001), laparoscopy approach (*p* = 0.006), aorta-related surgery (*p* = 0.017), Favier’s classification (*p* = 0.017), and transparietal colonic ischemia (*p* = 0.004) were statistically significant associated with mortality. After multivariate analysis, CCI (*p* = 0.024), APACHE II score (*p* = 0.001), Favier’s classification (*p* = 0.023) were independent predictors of mortality (Table 4). With regard to the evaluation of the cutoff point of APACHE II score for mortality, the area under the curve (AUC) was 86.0% (95% CI 77.9–94.1, *p* < 0.001). The cutoff point for the APACHE II score was 20.5 (optimal sensitivity and specificity were 66.7% and 93.3%, respectively) (Fig. 3).

**Table 4:** Predictive factors for mortality in patients with ischemic colitis

	Univariate	Multivariable
Year of surgery		
2011–2015	(Reference)	
2016–2020	0.90 (0.39–2.05, * p* = 0.792)	
Age	1.01 (0.99–1.04, * p* = 0.344)	
BMI	1.06 (0.95–1.17, * p* = 0.305)	
Gender		
Male	(Reference)	
Female	0.60 (0.26–1.40, * p* = 0.237)	
ASA		
2, 3	(Reference)	
4, 5	3.67 (1.48–9.11, * p* = 0.005)	0.20 (0.31–1.17, * p* = 0.074)
CCI	1.23 (1.05–1.44, * p* = 0.009)	1.29 (1.04–1.61, * p* = 0.024)
APACHE II	1.29 (1.17–1.43, * p* < 0.001)	1.25 (1.10–1.41, * p* = 0.001)
Laparoscopy	0.27 (0.11–0.68, * p* = 0.006)	0.61 ()0.16–2.33, * p* = 0.466)
Surgical delay	1.40 (0.61–3.22, * p* = 0.429)	
Aorta related surgery	4.67 (1.32–16.52, * p* = 0.017)	2.08 (0.40–10.72, * p* = 0.383)
Etiology of IC		
Postoperative IC	(Reference)	
Other etiology	0.75 (0.32–1.76, * p* = 0.51)	
Favier’s classification		
2	(Reference)	
3	7.48 (1.32–16.52, * p* = 0.017)	9.02 (1.35–60.37, * p* = 0.023)
Total colonic ischemia	7.31 (1.86–28.79, * p* = 0.004)	3.91 (0.55–27.80, * p* = 0.174)
Conversion	0.82 (0, 19–3.50, * p* = 0.786)	

BMI, Body mass index; ASA, American Society of Anesthesiologists; and APACHE, Acute Physiology and Chronic Health Evaluation

## Discussion

This propensity score matching comparison of emergent open versus laparoscopic colonic resection for IC confirmed that surgical site complications and duration of ventilation support were statistically significantly reduced after the laparoscopic approach. Of note, there was no statistically significant difference found in hospital stay, major complications, or mortality. After multivariate analysis, CCI (OR = 1.29, *p* = 0.024), APACHE II score (OR = 1.25, *p* = 0.001), and Favier’s classification (OR = 9.02, *p* = 0.023) remained statistically significant predictors of mortality (Table 4).

While randomized clinical trials are universally accepted as the gold-standard methodology for measuring the “causal” effects of management on outcomes [25], they are not always possible or adequately powered because they are time-consuming, costly, and may have ethical or practical constraints [26, 27]. Moreover, IC is rare; therefore, the accrual period for a randomized trial would be long and costly. Propensity score matching has gained in popularity in observational studies because potential biases in pretreatment characteristics between treatment groups are minimized [28].

Perioperative mortality can be as high as 60% after surgery and is most likely multifactorial, including degree of parietal involvement, location of ischemia, patient comorbidities, and physiological status [7]. However, after propensity-score matching, there was no statistically significant difference in patient variables thought to be associated with surgical outcomes in our study. Intraoperative findings (Favier’s classification, location of ischemia) were also included in propensity-score matching, even though these variables cannot be known precisely before operation, because they reflect severity of disease and technical difficulty. However, in 2011, Reissfelder et al. presented a risk score that predicts postoperative mortality of patients undergoing surgery for IC; perioperative variables included non-occlusive IC, acute renal failure, extent of bowel ischemia, serum lactate, and duration of catecholamine therapy [29]. As some of these factors were not included in our matching, they could eventually be part of a selection bias for treating patients with IC using an open or laparoscopic approach.

While laparoscopy is widely practiced in elective colorectal surgery, it is much less popular for emergency settings, although its feasibility and safety have been shown [11] and, ideally, should provide a smoother and less complicated postoperative course. Moreover, laparoscopy can be used with diagnostic intent in suspected IC, especially when colonoscopy is deemed dangerous [30] or for post-ischemic stenosis [15] and has the advantage of visualizing the entire colon, but is infrequently indicated for colectomy after acute ischemia and in particular in patients with IC. In the ACS-NSQIP database study, only 125 of 4548 (4.3%) colectomies for IC were performed laparoscopically [31]. Most published studies on the laparoscopic approach in patients with acute colorectal disease have focused on perforated diverticulitis, colorectal anastomosis leakage, or inflammatory colitis [12–14, 32–36]. All these studies showed that the laparoscopic approach could decrease the surgical site complication rate in the emergent setting but were not conclusive regarding overall complications, mortality, or hospital stay. This is in line with our results: The surgical site complication rate was lower in the laparoscopic group after propensity score matching. Furthermore, duration of ventilator support was shorter in the laparoscopic group after propensity score matching. Although there was a statistical trend toward shorter hospital stay, larger series are needed to demonstrate the advantages of the laparoscopy approach, and in particular in patients with severe disease or poor physiological status.

Symptoms of IC are often nonspecific, vague, and the diagnosis can be challenging at an early stage. In a review of 364 patients, peritoneal signs were present in only 7.4 percent of patients [1]. Furthermore, it is often difficult to identify symptoms in patients who are unconscious and debilitated, those in intensive care, or who are cognitively impaired, such as those with delirium or dementia. Therefore, IC must be suspected if a patient in an intensive care unit cannot tolerate a normal diet within a couple of days or has signs of infection. As ischemia in IC is initially mucosal, laparoscopic visualization of the serosa may seem normal, but conversely, as transmural ischemia can exist with minimal clinical signs, laparoscopic examination could be an early diagnostic tool for severe IC and lead to earlier surgical intervention.

When we compared the surgical delay between the laparoscopic and open groups, we found that the delay was longer in the open group than in the laparoscopic group (59.6% vs. 43.5%, *p* = 0.121; after propensity, 67.7% vs. 48.3%, *p* = 0.123). Although the difference was not statistically significant, this finding might further support the role of laparoscopy as an early diagnostic tool of the acute abdomen (EAES Symposium) [30, 37].

Our overall operation times (open: 200 min, laparoscopy: 180 min, *p* = 0.693) were comparable to those in another study [18]. However, in contrast to studies that focused on diverticulitis [12, 13], the operation time in the laparoscopy group was not statistically significantly longer than the open group. One reason might be that surgery for IC might have been more complex in the open group compared to laparoscopy group in spite of propensity score matching. Secondly, surgeons take more time to close a laparotomy compared to smaller laparoscopic surgical sites. Thirdly, all surgeries were performed by experienced hands in our institution. Based on our experience, we think laparoscopy should not prolong the operation time in the emergent cases and might even decrease the operation time in experienced hands.

Ischemic colitis is rare. The 2016 Premier Perspectives national inpatient database analysis indicated that only 12/945 (1.5%) laparoscopic colectomies were performed for noninfectious enteritis and colitis [38]. We agree with the authors that emergency laparoscopy has its place in emergency settings such as IC and there is a need to enlarge the indications [38]. Maggiori and Panis found that laparoscopic surgery for severe acute colitis was associated with a similar (or improved) short-term outcome compared with an open approach [39]. Sampietro et al. reported on 145 patients who underwent laparoscopic emergency subtotal colectomy for ulcerative colitis or Crohn’s disease stating that it was safe and feasible for acute severe colitis in IBD [40]. Our colectomy procedure is standardized, and all surgeons in our unit follow it. We believe that standardization of the surgical procedure could avoid mistakes especially when there are anatomical alterations and tissue inflammatory changes in the emergent setting. Finally, a laparoscopic second look is preferable to laparotomy. The timing of the second look is variable but usually should be within 72 h [41, 42].

While the causes that initiate ischemia may be variable, and even multiple, it is widely thought that these patients have vascular anomalies that enhance the onset: These can be constitutional or acquired. Among the former, there is no or a contentious connection between the middle colic and left colic arteries (Griffith’s point) in up to 48% of patients, or between the most distal sigmoid artery and the superior rectal artery (Sudeck’s point) in 5–15% of patients [43]. Previous colectomy or aortic surgery can further modify the vascular supply to the colon [43, 44]. Anastomosis after segmental colectomy for IC under these conditions might need specific maneuvers such as retroileal transmesenteric anastomosis (Toupet technique) or the Deloyers technique [45].

Previous studies have found various laboratory parameters to be associated with mortality, such as LDH > 450 U/l, blood urea nitrogen (BUN) (> 28 mg/dl), Hb < 12 g/dl, and hyponatremia (Na < 136 mEq/l) [46]. In our study, we used the APACHE II score to determine the general physical status of patients with IC. In multivariate regression analysis, APACHE II score (OR: 1.25, 95% CI 1.10–1.41, *p* = 0.001) was identified as an independent predictive factor for mortality. Furthermore, the APACHE II score model exhibited a high accuracy for the prediction of mortality, with an AUC of 0.86 (77.9–94.1, *p* < 0.001), comparable to that found by Peixoto et al. where the AUC was 0.89 [46]. In a large series of open colectomy, emergency surgery for IC was associated with high postoperative mortality [22]. In this study, preoperative lactates level, delay to surgery > 12 h, and the occurrence of postoperative acute kidney injury were independent predictors of postoperative mortality. Conversely, the specific cause of IC did not seem to impact postoperative mortality. These authors underlined the key role of prompt diagnosis and surgical intervention in the management of severe IC [22].

After propensity score matching, the conversion rate was 17/31 = 22.5%. The reasons for conversion were bowel distension, total colonic ischemia, diffuse fecal peritonitis, and/or severe adhesions. This is comparable to the literature on diverticular disease [13].

Our study has several limitations. Firstly, the sample size of this single institution case series was small (pre propensity, *n* = 96; post-propensity, *n* = 62). Secondly, as the decision to perform the operation laparoscopically was surgeon dependent, there may have been a selection bias even after propensity score matching. Thirdly, because of the retrospective design, it is not sure whether other factors may have influenced the postoperative outcomes, including intraoperative fecal spillage and bowel distension. Moreover, surgeon judgment intervened in the evaluation of the extent and degree of bowel ischemia to decide how much bowel should be resected and whether to perform primary anastomosis. In the future, intra-operative assessment by ICG might be an interesting avenue to explore [47]. Last, only known potential risk factors were matched. All confounding factors, those measured as well as those that are not measured, can only be eliminated by adequately conducted randomization. However, ischemic colitis is a rare disease, and therefore, a properly conducted and adequately powered randomized study would be difficult to perform. Finally, the inclusion time period of this study was long (2011–2020), and there have been many advances in surgical techniques and equipment, intensive care which may have impacted the surgical outcomes (such as operating time, postoperative complication rate, and hospital stay).

## Conclusion

Laparoscopic emergent colectomy for IC is feasible and safe, with fewer postoperative surgical site complications and reduced duration of ventilatory support, compared to laparotomy. Major complication and mortality rates remain high, essentially because of the severity of disease and patient status. We can postulate that the advantages of laparoscopic colectomy for patients requiring surgical intervention for ischemic colitis might reduce the morbidity and mortality in this setting, but this postulate needs to be proven in larger, and ideally, randomized studies.

## Data Availability

The data that support the findings of this study are available from the corresponding author, William Tzu-Liang Chen, on special request.
